# Selective pressures during chronic infection drive microbial competition and cooperation

**DOI:** 10.1038/s41522-019-0089-2

**Published:** 2019-06-07

**Authors:** Jiwasmika Baishya, Catherine A. Wakeman

**Affiliations:** Department of Biological Sciences, Texas Tech University, Lubbock, TX USA

**Keywords:** Microbiome, Evolution, Pathogens, Microbiome, Evolution

## Abstract

Chronic infections often contain complex mixtures of pathogenic and commensal microorganisms ranging from aerobic and anaerobic bacteria to fungi and viruses. The microbial communities present in infected tissues are not passively co-existing but rather actively interacting with each other via a spectrum of competitive and/or cooperative mechanisms. Competition versus cooperation in these microbial interactions can be driven by both the composition of the microbial community as well as the presence of host defense strategies. These interactions are typically mediated via the production of secreted molecules. In this review, we will explore the possibility that microorganisms competing for nutrients at the host–pathogen interface can evolve seemingly cooperative mechanisms by controlling the production of subsets of secreted virulence factors. We will also address interspecies versus intraspecies utilization of community resources and discuss the impact that this phenomenon might have on co-evolution at the host–pathogen interface.

## Introduction to life at the host–pathogen interface

### Pathogens must overcome host-imposed starvation

The host–pathogen interface is a battleground where reactive molecules and harsh conditions are produced by both the host and the pathogen. One of the harsh conditions faced by invading pathogens is the limitation of essential nutrients which are actively sequestered by the host immune response. These nutrients include metals such as zinc, manganese and iron, which serve as co-factors for several essential enzymes in pathogens and host cells.^1,2^ Sequestration of these metals and other nutrients by the host immune system during an infection is known as nutritional immunity.^2,3^ Nutritional immunity leads to starvation in pathogens and affects numerous cellular processes within them. Thus, pathogens are constantly at war with the host as well as other microbial species present at sites of infection to scavenge the resources required to establish infection and persist in a challenging host environment.

**Fig. 1:**
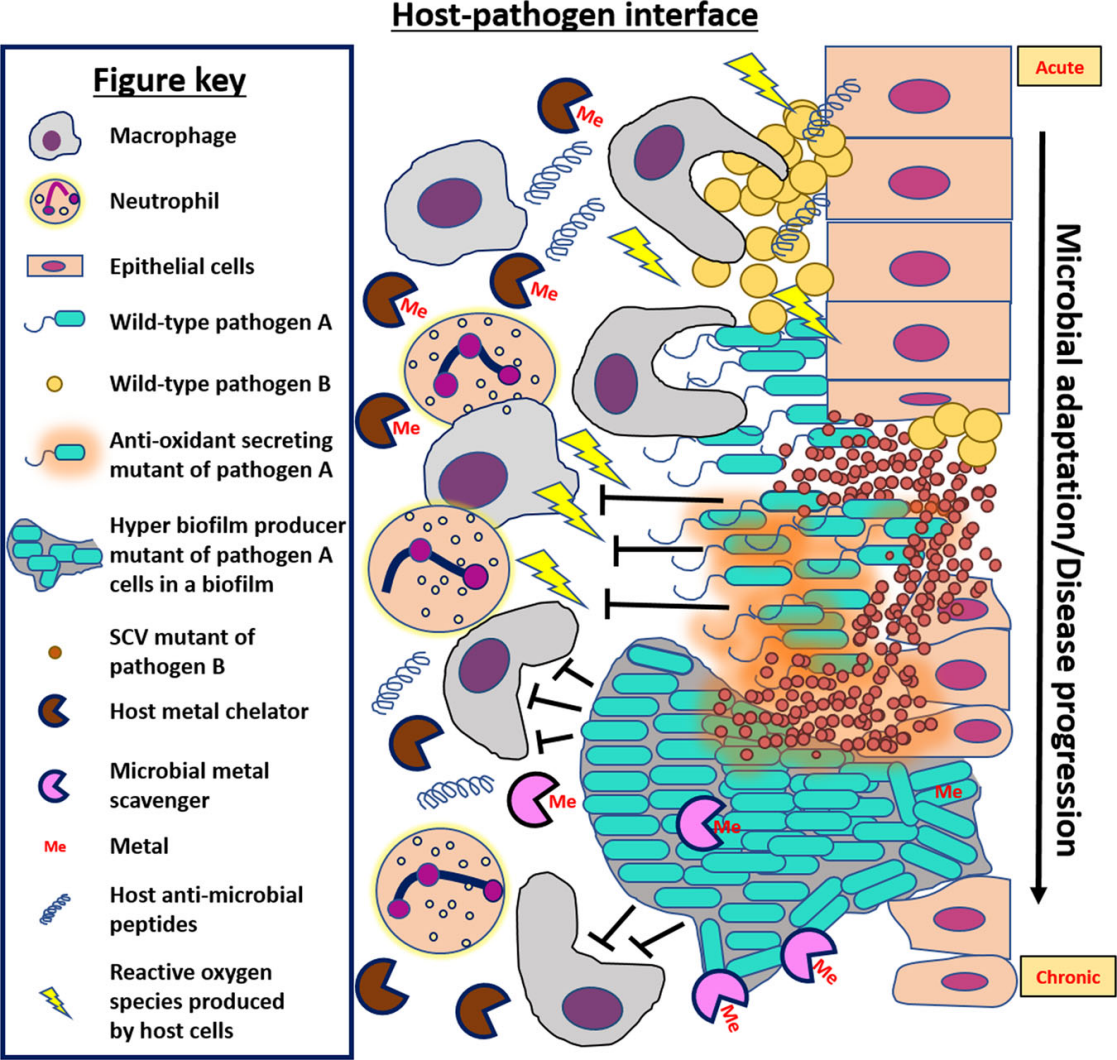
Selective pressures at the host–pathogen interface select for microbial adaptation and/or evolution. This figure depicts some of the possible stresses encountered by microbial pathogens while invading host tissues. For example, the innate immune system deploys phagocytes and neutrophils to target microbial pathogens at the site of infections. These cells can kill microbial cells by engulfing them and/or releasing antimicrobial molecules such as metal-binding proteins to sequester essential metals from pathogens, antimicrobial peptides such as LL-37, and reactive oxygen species. Pathogens like *Staphylococcus aureus* and *Pseudomonas aeruginosa* elicit physiological responses and/or evolve adaptations over the course of acute to chronic infection that allow them to circumvent these stresses and survive in spite of the presence of host offensive molecules. In this example, pathogen A is modeled after *P. aeruginosa* and pathogen B is modeled after *S. aureus*, but the disease progression may be applicable to other examples. Depicted here, initial infection of epithelial cells by *S. aureus* paves the way for opportunistic Gram-negatives such as *P. aeruginosa*. Microbial interactions of these species lead to formation of aminoglycosidic antibiotic resistant small colony variants (artistically depicted as smaller cells in the diagram) in *S. aureus* and might provide protection to *P. aeruginosa* from host immune insults. As the infection advances, the host–pathogen interface becomes populated by mutants that are selected for better survival such as mutants capable of dealing with host reactive oxygen species (such as pyomelanin producers depicted as releasing brown pigment) and mutants with increased biofilm formation to protect from phagocytosis and/or antimicrobial peptides such as LL-37

Many pathogens have been known to employ different metal acquisition systems to battle against host-imposed starvation and to outcompete nearby microbial species. For example, to combat the iron limitation mediated by host proteins such as lactoferrin, *Staphylococcus aureus* uses a combination of hemolysins and a high-affinity heme acquisition system to liberate heme from red blood cells for use as an iron source.^4^ A variety of mechanisms for circumventing this host-mediated starvation extends to many other microorganisms. Organisms such as the fungal pathogen *Cryptococcus neoformans* possesses enzymes such as ferric reductase that can acquire iron from the surrounding environment.^5^ Additionally, numerous organisms produce iron-scavenging siderophores^6,7^ (Fig. 1). Siderophores are small secreted molecules capable of binding iron (and sometimes other ions) with high affinity.^8^ While this struggle for nutrients intuitively creates competitive interactions, there is also evidence to support the existence of cooperative interactions designed to overcome such starvation. A potential example of this type of cooperation can be observed in the enhancement of the β-hemolytic activity of *S. aureus* in presence of other *Staphylococcus* species such as *S. epidermidis* as well as in the presence of certain *Corynebacterium* species.^9^ The increased hemolytic activity would presumably result in increased nutrient availability for each species at the host–pathogen interface, reducing the need for competition. *S. aureus* and *S. epidermidis* are both common components of the human microbiome so such *in vitro* findings support the idea that the evolution of microbial synergism intended to overcome host defenses could potentially occur during an infection.

### Mutagenic stressors are encountered at the host–pathogen interface

The stressors that microorganisms encounter at the host–pathogen interface expand beyond nutritional scarcity and include exposure to toxic molecules designed to kill microbial cells. These toxic molecules can be produced by both the host immune response and by other microorganisms (both commensals and pathogens) present within the host. Microorganisms occupying overlapping niches often attempt to outcompete each other to reduce competition in terms of limited nutrients and space. For example, *Pseudomonas aeruginosa* is known to outcompete co-infecting microorganisms like *Burkholderia cepacia* through the secretion of molecules such as pyocyanin that can generate reactive oxygen species (ROS).^10^ A study of *P. aeruginosa* and *Acinetobacter baumannii* showed pyocyanin leads to production of catalase and superoxide dismutase via quorum sensing in *A. baumannii*^11^ indicating that pyocyanin is capable of inducing oxidative stress in a range of microorganisms. Interestingly, *A. baumannii*’s response to this onslaught of ROS results in an increase in persister cell formation and antibiotic resistance, indicating that seemingly competitive interactions do not impact all microbial species equivalently.

In the same way that some microorganisms can utilize ROS to eliminate certain competitors, the host immune system possesses mechanisms to target invading pathogens through the release of these toxic molecules. Superoxides and other ROS produced by the host immune system can cause lethal damage to microbial macromolecules such as lipids, proteins and DNA. Hence, these are part of the first line of offensive molecules presented by host cells.^12,13^ Though many pathogens contain enzymes such as catalase and superoxide dismutase to counteract such oxidative stresses,^14,15^ often these enzymes require iron or manganese as key cofactors.^16^ Therefore, the nutritional immunity so often observed at the host–pathogen interface can compound these oxidative stresses by incapacitating the pathogens’ defense enzymes and increasing their susceptibility to ROS.^17^ While ROS-mediated DNA damage can kill microbial cells, it may also favor higher mutation rates which, when combined with the selective pressures at the host–pathogen interface, leads to great evolutionary potential.^18^

Other antimicrobial strategies employed by the host have similar mutagenic potential. For example, expression of antimicrobial peptide LL-37 has been described to increase mutation rates in invading pathogens. LL-37 is produced by host cells to disrupt bacterial cell membranes leading to cell lysis^19^ but recent studies have demonstrated that LL-37 can enter bacterial cells and induce mutation through erroneous replication by DNA polymerase IV.^20^ The increased mutation rates associated with LL-37 exposure in organisms like *P. aeruginosa* and *Escherichia coli* can even induce mutations involved in promoting resistance to the antibiotic rifampin.^19^

Another major stressor encountered by microorganisms infecting the human host is antibiotic treatment. Emergence of antibiotic resistance can be due to prolonged treatment, a major problem encountered in modern medicine. Current antibiotic treatments often fail when incomplete penetration of the drug into infectious sites creates exposure to subinhibitory concentrations and enables the emergence of partial resistance. Additionally, these subinhibitory concentrations of antibiotics can act as a signal for the microbial cells to alter their virulence and sometimes become more pathogenic.^21^ Such a microbial adaptation to stressors is not only detrimental to our ability to clear the infection, but it can also exacerbate the infection.^22–24^ This risk of adaptation to antibiotic treatment is greater in the presence of polymicrobial communities as a number of resistance genes counteracting many of the common microbially-derived antibiotics exists naturally within the microbial world. These resistance genes can sometimes be acquired by neighboring microbes. In fact, studies have shown that microbial genes conferring antibiotic-resistance in infected tissues can likely be acquired via horizontal gene transfer among co-infecting pathogens.^25^

**Fig. 2:**
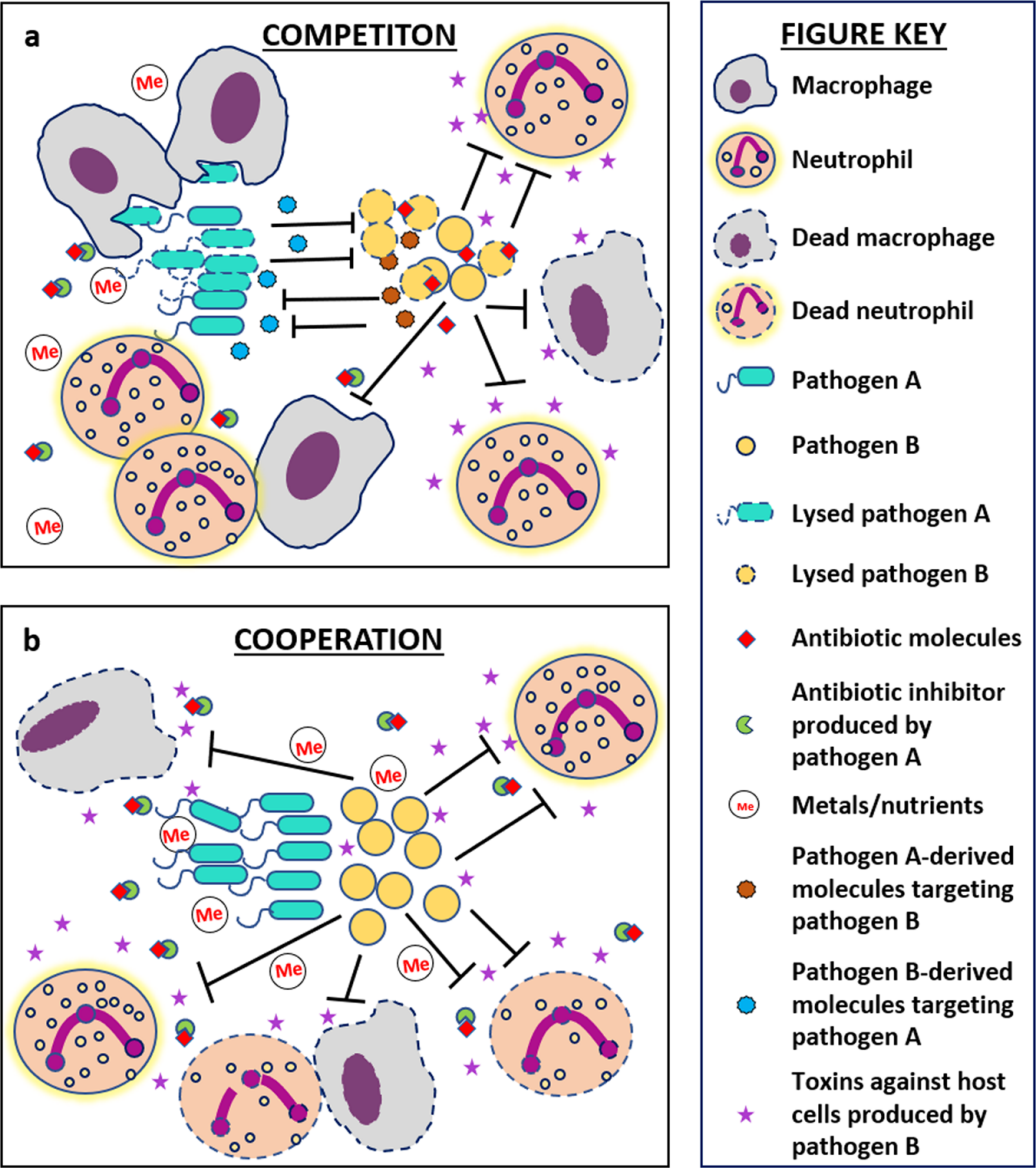
Competitive interactions versus cooperative polymicrobial interactions at the host–pathogen interface. This figure compares the potential costs and/or benefits experienced by microbial cells when they are interacting competitively versus cooperatively with other species inside a host. **a** Microbial cells exhibiting a competitive lifestyle must not only expend biosynthetic costs for production of competitive molecules to combat surrounding pathogens, but they must also independently deal with the onslaught of host-associated harsh conditions such as immune cells, antibiotic treatment, and nutrient limitation. **b** Microbial cells exhibiting a more cooperative lifestyle do not expend energy on antimicrobials and therefore may have more energy to expend on molecules designed to combat the host-associated harsh conditions. Additionally, they might also reap the benefits of the secreted molecules produced by their neighboring microbes to achieve greater levels of survival at the host–pathogen interface

From the decades of research performed on studies of the host–pathogen interface, we now understand that invading microbial species experience a number of selective pressures in this environment produced by the host and/or nearby microbial species. The fact that some of these pressures can induce genetic mutation greater than the basal level of mutation that occurs during replication creates a breeding ground for adaptive mutations that can enable survival in a harsh environment (Fig. 1). Development of such pathoadaptive mutations are now being studied in detail.^26,27^ Interestingly, a large number of these mutations are witnessed only in chronic infections and it is now well-known that majority of chronic infections contain polymicrobial biofilms.^28,29^ Therefore, we expect that future research will uncover pathoadaptive traits promoting either competition or cooperation between microbial species to enable survival within host niches. In this review we seek to explore the idea that mutations at the host–pathogen interface might favor cooperative interactions between otherwise competitive species at the face of hostile host environment. Cooperative interactions can reduce individual biosynthesis cost, allow persistent colonization leading to establishment of polymicrobial chronic infections, and potentially increase virulence of the pathogens as a community (Fig. 2). This type of cooperation could be attributed to the ability of neighboring microbes to exploit each other’s mechanisms for survival at the host–pathogen interface.

## Evolution in lung infections

### Biodiversity within lung infections

On the basis of standard culture techniques, lungs of healthy individuals were thought to be a sterile environment.^30^ However, the advent of metagenomic technologies has revealed that the healthy lung tissues contain microbial signatures from many species, mostly anaerobic bacteria like *Haemophilus*, *Prevotella spp*., and *Veillonella spp*., indicating that the healthy human lung might possess its own microbiome.^31,32^ The composition of this naturally occurring microbiota in healthy lung tissues is largely altered during infections.^33,34^ Difference in microbiome composition seen during different infections of the lung have been studied to understand their impact on host health. For example, an increase in population of microbes belonging to *Pseudomonas, Haemophilus* and *Stenotrophomonas spp* has been seen in the progression of chronic obstructive pulmonary disease.^35,36^ *Chlamydia pneumoniae* in lung tissues has been found in patients suffering from lung inflammations such as chronic bronchitis and asthma.^37,38^

Another disease that has been associated with dramatic shifts in the lung microbiome is cystic fibrosis. Cystic fibrosis is an autosomal recessive disorder caused by mutations in the *cftr* gene which encodes for the cystic fibrosis transmembrane regulator anion channel. Cystic fibrosis has many manifestations, one of which leads to accumulation of thick mucus in lung airways of the host and creates an environment ideal for microbial colonization.^39^ It was recently revealed that multiple microbial genera can occur in cystic fibrosis-associated lung infections as well. Different sequencing platforms have emphasized on different aspects of the microbial community composition in cystic fibrosis lungs. Some platforms detected presence of new species/strains in the samples whereas others identified the dominant species in the sample.^40^ Studies have shown that very early in life the lungs of cystic fibrosis patients tend to be colonized by common human commensals like *Streptococcus*, *Veillonella* and *Actinomyces*,^41–43^ but gradually they tend to be dominated by pathogenic species like, *P. aeruginosa*.^44,45^ Anaerobic bacterial species like *Gemella* spp.^46^ and fungal species like *Candida albicans* and *Aspergillus fumigatus* have also been identified in the lungs of cystic fibrosis patients.^45^ The composition of invading communities alters throughout the life of these patients and across different patients depending on host–pathogen and microbe-microbe interactions. While intervening host immune responses and antibiotic treatments can dramatically influence the microbial composition of infections,^25^ chronic infections can evolve stable and robust microbial systems that are often therapeutically resistant.^47,48^

### Competitive interactions inside the host

While invading host tissues, pathogenic microbes face stresses presented by nearby microbes in the form of antimicrobial molecules and competition for nutrients and space. These nearby microbes can either be members of the natural host microbiota (the commensals) or other co-infecting pathogens. Nevertheless, overcoming these stresses are crucial for surviving and colonizing in host tissues and hence competitive interactions are very commonly observed in polymicrobial infections. For example, *P. aeruginosa* and *S. aureus* are commonly seen to co-colonize cystic fibrosis-associated lung airways.^49^ These microbes must compete for iron, which is sequestered by the host’s nutritional immunity. Therefore, iron depletion can act as a global regulatory signal for microbes such as *P. aeruginosa* to turn on genes for the synthesis of competitive molecules enabling the scavenging of this precious resource.^50^

In order to do so some microorganisms steal or “pirate” iron using siderophores of other pathogens, while others directly lyse cells to acquire the stored intracellular iron.^8^ Siderophores can be pirated through mutations in an organisms’ siderophore transport machinery to enable recognition of other siderophores. In order to prevent such activities, microorganisms often modify their siderophores structurally to increase specificity. Competing organisms can acquire mutations in their acquisition systems to recognize the new siderophore modifications giving rise to an evolutionary battle.^2,8^ A recent study also showed that under low iron conditions such as those occurring inside host peritoneum, pathogenic *P. aeruginosa* lyses *S. aureus* and *S. pneumoniae* cells to acquire their stored iron.^51^ Iron depletion in cystic fibrosis lungs also shows a similar phenomenon where antimicrobials such as 2-alkyl-4(1H)- quinolones are secreted by *P. aeruginosa* to suppress the growth of *S. aureus*.^52^

In cases of infections comprising *P. aeruginosa* and *C. albicans*, anti-microbials known as phenazines are produced by the bacterium which inhibit fungal biofilm formation by preventing the latter’s yeast to filament transition.^53^ Phenazines such as pyocyanin suppress growth of *C. albicans* on non-fermentable carbon sources by restricting respiration via limited oxygen availability. This interaction represents a competitive approach taken by *P. aeruginosa* via which it can utilize the products of fermentation for enhancing its growth and survival without actually having to produce them.^54^ However, polymicrobial interactions in the presence of phenazines do not always result in *P. aeruginosa* being the clear winner of the competition. For example, *A. fumigatus* possesses a unique ability to process and transform the *P. aeruginosa*-derived phenazines into unique molecules and can serve as interspecies signals.^55^ The presence of these signals activates a series of responses in *A. fumigatus* including oxidative stress responses as well as the induction of siderophore production, which might provide the fungus with its own competitive strategies against *P. aeruginosa*.^55,56^ Thus, a tug-of-war exists between microbial species to acquire essential nutrients. Interactions of such kind depict an ongoing molecular arms race within co-evolving pathogenic species.^8^

### Individual adaptations to the lung environment

Despite the severe competitive pressure imposed by resource scarcity (such as transition metals) at the host–pathogen interface, a large number of the pathoadaptive mutations observed during chronic infections are not aimed at increasing microbial competitive strategies. Instead these adaptations are more focused on protective strategies such as evading the immune response and/or therapeutic intervention. This phenomenon indicates that the pressures faced as a result of the active immune response and antibiotic treatment may be even more influential to the evolution of pathogens than the pressures imposed by competing microbes. A plethora of adaptive changes have been described in *S. aureus* (Fig. 1), which is an early pathogenic colonizer of chronic lung infections.^57,58^ Biofilm formation,^59^ production of capsular polysaccharides,^60^ switching to small colony variant (SCV) phenotype,^61^ evolution of methicillin-resistant *S. aureus* strains (MRSA)^62^ and the recent emergence of vancomycin-resistant *S. aureus* strains (VRSA)^63^ are a few of such adaptations. These adaptive changes impart therapeutic resistance to *S. aureus* cells and facilitate recurrent infections in patients despite undertaking antibiotic treatments. This antibiotic resistance can be either due to acquisition of antibiotic-resistance genes as in the case of MRSA or due to absence of a proton motive force essential to pump antibiotics inside the microbe as in the case of SCVs.^64^ Biofilms are often associated with antibiotic tolerance simply due to limited diffusion through their thick biomass. Additionally, *S. aureus* can evolve mutations to survive in the presence of the competitive molecules produced by neighboring organisms such as *P. aeruginosa*.^65^

Many individual adaptations have also been observed in *P. aeruginosa* cells colonizing lung airways of adult cystic fibrosis patients^66^ (Fig. 1). For example, a study conducted whole-genome sequencing to compare gene sequences of two isolates of *P. aeruginosa*, one from an early (6 month) cystic fibrosis -infection sample and the other from a late (8 year) cystic fibrosis -infection sample. In total, 68 mutations were detected.^67^ Most mutations, in both the isolates, were present in the *mexZ* gene, a negative regulator of the MexXY-OprM multidrug efflux pump. Mutation in this gene confers resistance against aminoglycoside antibiotics like tobramycin.^67^ Another mutation was detected in the tyrosine catabolic pathway that leads to overproduction of pyomelanin, a molecule which provides resistance against oxidative stress.^18,68^ In other chronic cystic fibrosis lung isolates, mutations leading to overproduction of alginate have been commonly witnessed. Alginate is produced as a structural component of their biofilm extracellular polymeric substances (EPS), thus imparting antibiotic resistance and protection against ROS to microbial cells.^69–71^ A loss of function in the *mucA* gene leads to overproduction of alginate in *P. aeruginosa*.^66,72^ Some studies also show that LL-37, at sub-inhibitory levels, promotes polysaccharide production in organisms like Group A *Streptococci*, *E. coli and P. aeruginosa*.^19,73^ The range of microbial adaptive strategies to the harsh environment of the cystic fibrosis lung is wide, even within the same patient. For example, isolates of *P. aeruginosa* from different lobes of the same lung were found to have completely different antibiotic susceptibilities and virulence levels.^74^

### Potential for evolution of cooperation to exploit intra- and interspecies adaptations

Microbial communities isolated from host tissues containing different strains and species exhibit diverse adaptive mutations. While the majority of these adaptations are likely to only benefit the adapted organism, some of these adaptations involve the secretion of molecules designed to make the host environment more hospitable (Figs 1 and 2). Theoretically, these secreted molecules could also be exploited by surrounding microorganisms (Fig. 2). For example, mixed communities of *P. aeruginosa* mucoid strains and nonmucoid revertants show enhanced resistance to both LL-37 and H_2_O_2_ produced by the host immune response. Alginate produced as a part of the EPS of mucoidy cells protects this community from LL-37 mediated killing and catalase produced by nonmucoidy cells protects the community from H_2_O_2._ The mucoid phenotype in *P. aeruginosa* is correlated with deteriorating cystic fibrosis lung infection and therefore this intraspecies mutualism indicates a selective benefit in co-existence.^69,71^ A study has shown that *P. aeruginosa* isolated from chronically infected CF patients exhibit parallel intraspecies mutualism where isogenic strains, auxotrophic for different nutrients, show complementation and reduced virulence.^75^

Similar intraspecies interactions have been seen in case of *S. aureus*. Growth-deficient mutants Δ*hemB* and Δ*menB* have impaired growth and exhibit the SCV phenotype due to mutations in their heme and menaquinone biosynthetic pathways, respectively. These individual mutants “rescue” each other’s growth deficiencies via cross-feeding of heme and menaquinone when present together and achieve recovery of fitness and infectivity similar to the wild-type.^76^ Interestingly, this same study observed similar cooperative behavior occurring between *S. aureus* SCVs and other species isolated from cystic fibrosis infections, demonstrating that interspecies cooperation at the host–pathogen interface is possible.

In addition to the exchange of beneficial molecules, studies have demonstrated interspecies cooperation as a result of repression of competitive molecules produced by pathogenic microbes. An example of this type of cooperation is the reduction of pyocyanin production by multidrug resistant strains of *P. aeruginosa* isolated from chronically infected tissues. Pyocyanin, a bluish pigment, is a major virulence factor of *P. aeruginosa* and loss of it could enable this microbe to pursue non-competitive interactions with nearby species.^77^ In fact, it has been shown that the presence of calprotectin, an innate immune protein, in the host environment leads to a repression of pyocyanin and alkyl quinolones and promotes co-infection in the murine lung with *S. aureus*.^78^ This phenomenon is counterintuitive as the signal for repression of these molecules is zinc starvation induced by calprotectin and in this starved environment, an increase in the production of competitive molecules would be more expected. Mutations leading to alginate overproduction in *P. aeruginosa* also promotes co-infection with *S. aureus* by reducing production of the anti-staphylococcal molecules by *P. aeruginosa*.^79^

Potential advantages conferred by decrease in competitive molecules might be associated with the ability of *P. aeruginosa* to exploit the anti-immunity factors that *S. aureus* can produce. For example, *S. aureus* cells can assist in colonization of host tissues by Gram-negative bacteria. Virulence factors of *S. aureus* such as α-toxin allows proliferation and dissemination of Gram-negatives such as *P. aeruginosa* and *Klebsiella pneumoniae* by counteracting components of the human immune response.^80^ *S. aureus* also facilitates survival of *lasR* mutants of *P. aeruginosa* commonly found in cystic fibrosis patients by detoxifying surrounding nitric oxide released by host immune cells.^81^ In addition to *S. aureus* conferring benefits to *P. aeruginosa*, there are also instances of the reciprocal interaction. For example, protection of cystic fibrosis isolates of *S. aureus* cells from multiple antibiotics have also been reported recently.^82^ These cells were extracted from patients co-infected with *P. aeruginosa*. When present together, 4-hydroxy-2-heptylquinoline-N-oxide (HQNO) produced by *P. aeruginosa* cells inhibited respiration in *S. aureus*. While the presence of HQNO and associated reduction in respiration reduces the growth rate of *S. aureus*, it also protects the *S. aureus* cells from aminoglycosidic antibiotics.^82^ HQNO is produced by *P. aeruginosa* in its natural habitat primarily to slow down the growth of competitor cells in the vicinity^83,84^ but in host tissues the presence of HQNO may actually provide a benefit to *S. aureus* by promoting antibiotic tolerance.

This potential benefit of co-infection may extend past *S. aureus* and *P. aeruginosa* polymicrobial communities. For example, *S. aureus* cells have also been known to become tolerant to vancomycin in presence of the fungus, *C. albicans*.^85^ When present together *S. aureus* uses *C. albicans* cells as a substratum to form biofilms upon an existing biofilm. Also, the extracellular DNA component of *C. albicans*’s EPS promotes stability of *C. albicans*-*S. aureus* dual-biofilms and augments resistance of the former to anti-fungal molecules such as miconazole.^86^ In case of *Streptococcus agalactiae*, facilitation of growth via respiration by *Lactococcus lactis* has been studied as another example of interspecies cooperation. *L. lactis* synthesizes demethylmenaquinone in presence of heme which can be used by *S. agalactiae* to activate respiratory metabolism and enhance virulence in human blood.^87^ Respiration has been shown to be crucial in *S. agalactiae*’s virulence and thus, infections caused by *S. agalactiae* worsen in presence of *L. lactis*. Thus, we see there is significant potential for microbial cooperation among otherwise competing microbial species when present in polymicrobial contexts such as those observed in chronically infected host tissues. This phenomenon indicates that maybe tensions at the host–pathogen interface are driving an evolutionary shift in these species to become cooperative as opposed to being competitive for securing a long-term infection as a community. This type of cooperation can be corroborated by the study that showed that *P. aeruginosa* can protect members of the polymicrobial consortium of the cystic fibrosis lung namely, *S. aureus*, *Inquilinus limosus* and *Stenotrophomonas maltophilia* from antibiotics such as ciprofloxacin.^88^

Considering such discoveries, we hypothesize that due to the presence of the intensified pressures at the host–pathogen interface, the host versus microbe molecular arms race takes precedence over the natural microbe versus microbe race and opens doors to symbiotic or cooperative interactions with fellow pathogens and/or commensals. This phenomenon could be mediated by the fact that cooperative interactions can aid microbes to survive by joining forces against the host immune system and waging the battle together by exploiting shared resources, instead of expending energy in the production of antimicrobial compounds against each other (Fig. 2).

## Applicability to other host niches

While this review is mostly focused on polymicrobial interactions within the lung, similar adaptations are likely to occur in other host-relevant niches. Microbial cooperation has been well characterized as occurring in human oral cavities and is likely also occurring in the context of chronic wound infections. Co-evolution has been very well depicted in the communities shaping these two host environments. The following sections describe instances where microorganisms of the oral cavity or the chronic wound environment display cooperation over competition.

### Oral cavity

The healthy human oral cavity harbors as many as 200 different bacterial species^89,90^ along with viruses and fungi. These species form inter-connected networks to survive in the lotic environment of the mouth. Cooperation among these microbes is seen in terms of co-adhesion or co-aggregation of cells that attach to each other for establishing colonization onto oral surfaces like the gumline, tooth enamel and the sub-gingival cavities. Early colonizers such as *Streptococcus gordonii*, *Propionibacterium acnes, Haemophilus parainfluenzae* and *Prevotella loescheii* initiate colonization by binding to complementary salivary receptors such as sialylated mucins, proline-rich proteins and α-amylase present on the tooth surface. These cells coaggregate with late colonizers such as *Aggregatibacter actinomycetemcomitans* and *Treponema denticola* to promote polymicrobial biofilm formation.^91,92^ Sometimes, intermediary species even “bridge” the early and later colonizers. For example, *Fusobacterium nucleatum* expresses at least one adhesin that recognizes early colonizer *Streptococci spp*. and a galactose-specific lectin that interacts with late colonizer, *Porphyromonas gingivalis*.

Although a large portion of the oral microbiome consist of commensals, a subset of these are opportunistic pathogens.^93^ These pathogens can cause oral cavity infections such as periodontitis that have been widely studied for cooperative interactions among causative microorganisms.^94^ *P. gingivalis*, *T. denticola* and *Tannerella forsythia* are known to be the primary causative pathogens of oral periodontitis^95^ though recently many anaerobic bacterial species have been discovered to be associated as well.^93^ Periodontitis results from dysbiosis that changes the microbial composition of the oral cavity from a healthy to a diseased state.^96^ In particular, *P. gingivalis* has been revealed as a keystone pathogen driving the dysbiosis associated with chronic periodontitis through the production of a suite of virulence factors capable of benefiting itself as well as the surrounding diseased community.^97^ Additionally, *P. gingivalis* is known to release eDNA that can be up taken by microorganisms inducing adaptive mutations in them.^98^ This process can make neighboring pathogens immune to known antibacterial molecules and increase pathogenicity of the community. Other studies have revealed that periodontal pathogens such as *F. nucleatum and A. actinomycetemcomitans* generate short-chain fatty acids (SCFAs) such as butyric acid which aid in disease progression possibly via facilitating pathogen colonization.^99–101^ SCFAs induce ROS production and inflammation in the gingival tissues leading to growth inhibition of gingival epithelial cells that serves as a barrier against pathogenic invasion and impair healing.^99,100^

Synergistic interactions between early and late colonizers have been shown to enhance virulence of opportunistic oral pathogens such as *A. actinomycetemcomitans*. For example, during a co-culture of *A. actinomycetemcomitans* and the oral commensal *S. gordonii*, the latter increased the virulence of the former by providing it with l-lactate, a carbon source necessary for establishing robust infection in the gum subgingival crevice of human oral cavity.^102^ l-lactate is primarily produced by *S. gordonii* cells and is made available to *A. actinomycetemcomitans* via metabolic cross-feeding. *S. gordonii* cells also produce inhibitory levels of H_2_O_2_ to trigger catalase mediated break-down by *A. actinomycetemcomitans*. Production of such ROS augments bioavailability of oxygen during an infection, allowing *A. actinomycetemcomitans* cells to shift from a primarily fermentative to a respiratory metabolism which in turn enhances its growth and persistence.^103^ Additionally, this catalase produced by *A. actinomycetemcomitans* protects anaerobic pathogen *P. gingivalis* from ROS in the oral cavity and aids it to colonize oral surfaces other than the dental pockets where oxygen is limited.^104^

In a study about interactions of oral pathogens, intergeneric co-aggregation was shown in mixed-species communities of *Streptococci* and *Veillonellae spp*. during formation of early dental plaque. Lactic acid produced by *Streptococci* is cross fed to *Veillonellae* cells, where it serves as a carbon source and establishes metabolic interaction between the two microbial species. Biofilm community obtained from a retrievable human enamel surface revealed two *Streptococci*, *S. oralis* and *S. gordonii*, and an uncultivated *Veillonellae spp*. as members of such mixed-species colonies.^105^ Another study that investigated the interactions between the fungus *C. albicans* and three commensal *Streptococci spp*. of the human oral cavity showed that *Streptococci* cells displayed poor biofilm formation on abiotic or mucosal surfaces by itself but formed robust biofilms in presence of the fungus. On the other hand, these *Streptococcal spp*., such as *S. oralis*, enhanced the ability of *C. albicans* to invade oral and esophageal mucosa.^106^ Thus, co-adhesion and co-aggregation facilitated metabolic interdependencies have been very well established in the human oral cavity. These dependencies play important roles in determining the chronology of colonization in the oral cavity.

### Chronic wounds

Chronically infected sites such as chronic wounds are also likely to experience similar levels of microbial interactions as in the oral cavity. Chronic wounds can be an outcome of burns, surgical site infections, and/or diabetic foot ulcers which are often slow-healing or non-healing.^107^ A study of 30 human wounds revealed the presence of 106 different bacterial genera, large fractions of which were strict and facultative anaerobes. Such research indicates the presence of a diverse microbial community in chronically wounded tissues.^108^

Recent studies have shown enhanced antibiotic tolerance in polymicrobial communities of chronically wounded tissues. For example, one study showed that chronic wounds colonized with biofilms of three bacterial species- *S. aureus*, *P. aeruginosa* and *Clostridium perfringens*, were found to be more tolerant to treatment than single-species wound infection.^109^ Another study aimed at understanding interspecies interaction between *S. aureus*, *P. aeruginosa*, *Enterococcus faecalis* and *Finegoldia magna* under in vivo mouse wound conditions, showed that the presence of a microbial consortium enabled growth of the obligate anaerobe *F. magna*.^110^ As in other host-associated niches, microbial cooperation in the form of cross-feeding appears to play an important role in establishment of the wound polymicrobial community.^111^ For example, wound-associated *S. aureus*, *E. coli*, and *K. pneumoniae* can provide molecules such as heme, menaquinone, and succinate to pathogenic *Prevotella* and *Porphryomonas spp*.^112^ In another study, augmentation of *E. coli* biofilms in mouse wound infection model has been shown to occur as a result of ornithine production by neighboring *E. faecalis*.^111^ Presence of ornithine modulates the surrounding environment of *E. coli* in ways that favor metabolic pathways leading to siderophore biosynthesis. In addition to nutrient complementation mediated by the surrounding microbial consortium, mice with mixed-species biofilm infections displayed wound healing impairment and increased antimicrobial tolerance as compared to mice infected with single species of these bacteria.^113^ Thus, synergistic interactions between different bacterial species in wounds confer the participating cells with benefits such as antibiotic tolerance and reduced interspecies competition. Additionally, the chronic wounds create an environment that promotes a reduction in competitive molecules produced by *P. aeruginosa* to enable this synergism. In addition to being able to suppress its antimicrobial arsenal in the presence of the innate immune protein calprotectin,^78^ *P. aeruginosa* exhibits reduced anti-staphylococcal activity in the presence of the abundant host protein albumin.^114^

## Potential for therapeutic targeting

Infectious polymicrobial communities are often found to be more resistant to antibiotics than their mono-culture counterparts.^82,88^ Members of these polymicrobial communities are actively interacting with one another and building a network of interactions. These interactions are dictated by adaptive mutation which may initially seem detrimental but appear useful in context of the community. Here we have discussed several examples of microbial infections where we see how polymicrobial interactions and shifts in community composition modulate pathogenicity and antimicrobial susceptibility of the pathogens. Over the course of chronic infection, the microbial community composition tends to fluctuate, beginning with transient colonization and transforming into a highly pathogenic and persistent infection.^115^ An understanding of these shifts is important for designing therapeutics because of the metabolic differences exhibited by these communities. For example, in infections such as the ones caused by both *P. aeruginosa* and *S. aureus*, aminoglycosides might not be the most effective therapeutic due to the increased aminoglycoside resistance conferred to *S. aureus* by the presence of *P. aeruginosa*.^82^ In addition to being influenced by neighboring communities, microbial physiology is also driven by the overall community structure.^116^ This community structure introduces nutrient gradients within the community that drive physiological differentiation of microbes which can alter antibiotic susceptibility.^117^ Efforts are being made to better understand the core metabolism of chronic infectious communities as well as the biogeochemical forces influencing microbial physiology in order to identify the most effective drug targets.^34^

While polymicrobial interactions may provide complications for traditional therapeutics, it is also possible that these interactions could provide new drug targets. For example, if the structure of the microbial community is important for its persistence, mechanisms that disrupt the biogeography of the infection could prove useful.^116^ Mechanisms to target and disrupt structural components of biofilms are currently being pursued by many research groups.^118–120^ Additionally, if these complex microbial communities are relying on secreted shared resources for survival, targeting of these resources might severely impact both the producer microbes as well as beneficiary microbes in the vicinity.

### Reporting Summary

Further information on research design is available in the Nature Research Reporting Summary linked to this article.

### Disclaimer

The content is solely the responsibility of the authors and does not necessarily represent the official views of the National Institutes of Health.

## Supplementary information

reporting summary checklist

## Supplementary information

**Supplementary information** accompanies the paper on the *npj Biofilms and Microbiomes* website (10.1038/s41522-019-0089-2).
